# Bis[4-(dimethyl­amino)pyridinium] tetra­bromidobis(4-methyl­phen­yl)stannate(IV)

**DOI:** 10.1107/S1600536809030323

**Published:** 2009-08-08

**Authors:** See Mun Lee, Kong Mun Lo, Hapipah Mohd Ali, Ward T. Robinson

**Affiliations:** a Department of Chemistry, University of Malaya, 50603 Kuala Lumpur, Malaysia

## Abstract

In the title compound, (C_7_H_11_N_2_)_2_[SnBr_4_(C_7_H_7_)_2_], the tetra­bromidobis(4-methyl­phen­yl)stannate(IV) anion possesses a centre of inversion located at the Sn^IV^ atom. In the crystal structure, two inversion-related cations are linked to the anion *via* weak N—H⋯Br hydrogen bonds.

## Related literature

For related crystal structures, see Lo & Ng (2009 ▶); Koon *et al.* (2009 ▶); Yap *et al.* (2008 ▶).
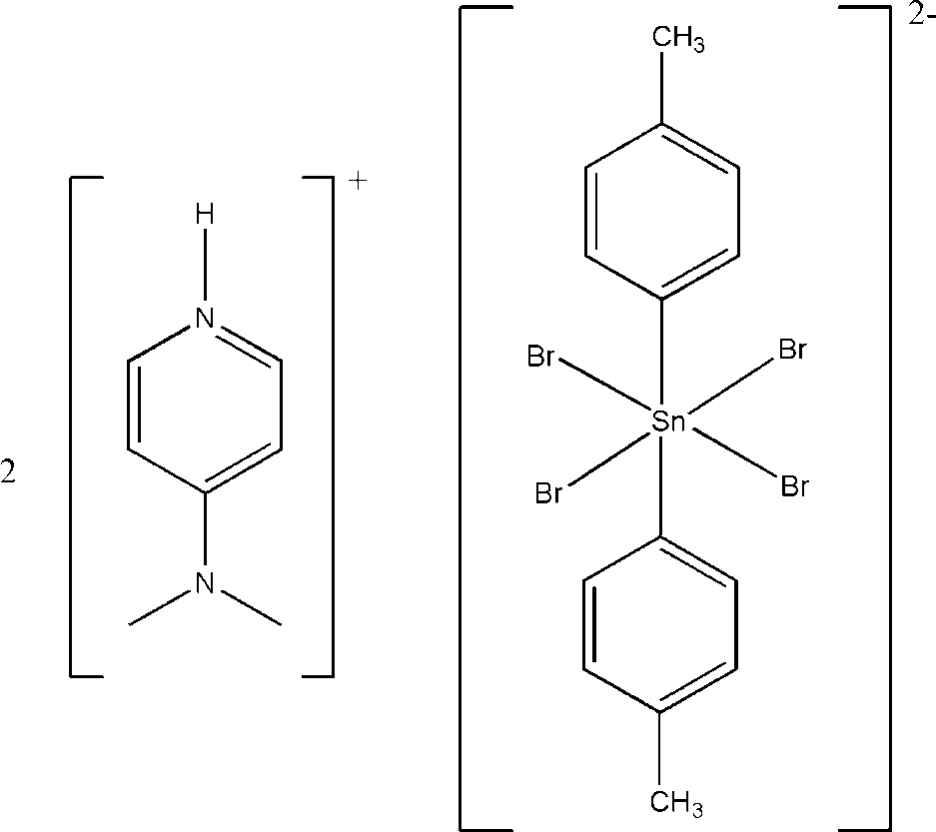


## Experimental

### 

#### Crystal data

(C_7_H_11_N_2_)_2_[SnBr_4_(C_7_H_7_)_2_]
*M* *_r_* = 866.94
Monoclinic, 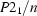

*a* = 10.2178 (3) Å
*b* = 10.4808 (3) Å
*c* = 14.5833 (3) Å
β = 95.063 (1)°
*V* = 1555.64 (7) Å^3^
*Z* = 2
Mo *K*α radiation
μ = 5.98 mm^−1^
*T* = 100 K
0.35 × 0.30 × 0.22 mm

#### Data collection

Bruker APEXII CCD area-detector diffractometer
Absorption correction: multi-scan (*SADABS*; Sheldrick, 1996 ▶) *T*_min_ = 0.229, *T*_max_ = 0.353 (expected range = 0.174–0.268)
11555 measured reflections
3569 independent reflections
3225 reflections with *I* > 2σ(*I*)
*R*_int_ = 0.019

#### Refinement

*R*[*F* ^2^ > 2σ(*F* ^2^)] = 0.018
*wR*(*F* ^2^) = 0.045
*S* = 1.05
3569 reflections
172 parameters
H-atom parameters constrained
Δρ_max_ = 0.44 e Å^−3^
Δρ_min_ = −0.45 e Å^−3^

### 

Data collection: *APEX2* (Bruker, 2008 ▶); cell refinement: *SAINT* (Bruker, 2008 ▶); data reduction: *SAINT*; program(s) used to solve structure: *SHELXS97* (Sheldrick, 2008 ▶); program(s) used to refine structure: *SHELXL97* (Sheldrick, 2008 ▶); molecular graphics: *SHELXTL* (Sheldrick, 2008 ▶); software used to prepare material for publication: *publCIF* (Westrip, 2009 ▶).

## Supplementary Material

Crystal structure: contains datablocks I, global. DOI: 10.1107/S1600536809030323/lh2842sup1.cif

Structure factors: contains datablocks I. DOI: 10.1107/S1600536809030323/lh2842Isup2.hkl

Additional supplementary materials:  crystallographic information; 3D view; checkCIF report

## Figures and Tables

**Table 1:** Hydrogen-bond geometry (Å, °)

*D*—H⋯*A*	*D*—H	H⋯*A*	*D*⋯*A*	*D*—H⋯*A*
N1—H1⋯Br1^i^	0.88	2.75	3.448 (2)	138
N1—H1⋯Br2^i^	0.88	2.94	3.517 (2)	125

Symmetry code: (i) 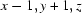.

**Fig. 1.**
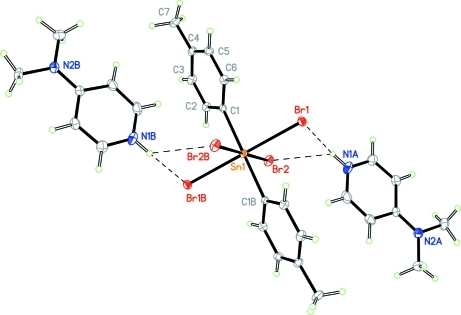
The molecular structure of the title compound with 50% probability displacement ellipsoids and the atom numbering. Hydrogen atoms are drawn as spheres of arbitrary radius and dashed lines indicate hydrogen bonds (symmetry codes: (A) 1+x, -1+y, z and (B) 2-x, -y, -z).

## supplementary crystallographic information

### Comment

The molecular structure of the title compound is shown in Fig. 1.

### Experimental

Tetra(4-methylphenyl)tin (0.49 g, 1 mmol) and 4-dimethylaminopyridine hydrobromide perbromide (0.40 g, 1 mmol) were dissolved in absolute ethanol (25 ml) and refluxed for six hours. The solution was filtered and colourless crystals were isolated upon cooling to room temperature.

### Refinement

Hydrogen atoms were placed at calculated positions (C–H 0.95 to 0.98 Å) and were treated as riding on their parent carbon atoms, with *U*(H) set to 1.2–1.5 times *U*(C,*N*). N—H was refined and placed in the calculated position of N—H 0.88 ± 0.01 Å.

### Crystal data

**Table:**

(C_7_H_11_N_2_)_2_[SnBr_4_(C_7_H_7_)_2_]	*F*(000) = 844
*M**_r_* = 866.94	*D*_x_ = 1.851 Mg m^−^^3^
Monoclinic, *P*2_1_/*n*	Mo *K*α radiation, λ = 0.71073 Å
Hall symbol: -P 2yn	Cell parameters from 6698 reflections
*a* = 10.2178 (3) Å	θ = 2.3–30.5°
*b* = 10.4808 (3) Å	µ = 5.98 mm^−^^1^
*c* = 14.5833 (3) Å	*T* = 100 K
β = 95.063 (1)°	Block, colourless
*V* = 1555.64 (7) Å^3^	0.35 × 0.30 × 0.22 mm
*Z* = 2	

### Data collection

**Table:**

Bruker APEXII CCD area-detector diffractometer	3569 independent reflections
Radiation source: fine-focus sealed tube	3225 reflections with *I* > 2σ(*I*)
graphite	*R*_int_ = 0.019
ω scans	θ_max_ = 27.5°, θ_min_ = 2.3°
Absorption correction: multi-scan (*SADABS*; Sheldrick, 1996)	*h* = −13→10
*T*_min_ = 0.229, *T*_max_ = 0.353	*k* = −13→8
11555 measured reflections	*l* = −18→18

### Refinement

**Table:**

Refinement on *F*^2^	Primary atom site location: structure-invariant direct methods
Least-squares matrix: full	Secondary atom site location: difference Fourier map
*R*[*F*^2^ > 2σ(*F*^2^)] = 0.018	Hydrogen site location: inferred from neighbouring sites
*wR*(*F*^2^) = 0.045	H-atom parameters constrained
*S* = 1.05	*w* = 1/[σ^2^(*F*_o_^2^) + (0.0246*P*)^2^ + 0.5727*P*] where *P* = (*F*_o_^2^ + 2*F*_c_^2^)/3
3569 reflections	(Δ/σ)_max_ = 0.001
172 parameters	Δρ_max_ = 0.44 e Å^−^^3^
0 restraints	Δρ_min_ = −0.45 e Å^−^^3^

### Special details

**Table:**

Geometry. All e.s.d.'s (except the e.s.d. in the dihedral angle between two l.s. planes) are estimated using the full covariance matrix. The cell e.s.d.'s are taken into account individually in the estimation of e.s.d.'s in distances, angles and torsion angles; correlations between e.s.d.'s in cell parameters are only used when they are defined by crystal symmetry. An approximate (isotropic) treatment of cell e.s.d.'s is used for estimating e.s.d.'s involving l.s. planes.
Refinement. Refinement of *F*^2^ against ALL reflections. The weighted *R*-factor *wR* and goodness of fit *S* are based on *F*^2^, conventional *R*-factors *R* are based on *F*, with *F* set to zero for negative *F*^2^. The threshold expression of *F*^2^ > σ(*F*^2^) is used only for calculating *R*-factors(gt) *etc*. and is not relevant to the choice of reflections for refinement. *R*-factors based on *F*^2^ are statistically about twice as large as those based on *F*, and *R*- factors based on ALL data will be even larger.

### Fractional atomic coordinates and isotropic or equivalent isotropic displacement parameters (Å^2^)

**Table:**

	*x*	*y*	*z*	*U*_iso_*/*U*_eq_	
Sn1	1.0000	0.0000	0.0000	0.01042 (5)	
Br1	0.951470 (19)	−0.180492 (17)	0.131083 (12)	0.01383 (5)	
Br2	1.261950 (18)	−0.018292 (18)	0.055861 (14)	0.01597 (6)	
N1	0.24150 (18)	0.65685 (17)	0.11335 (13)	0.0237 (4)	
H1	0.2028	0.7281	0.1280	0.028*	
N2	0.43055 (16)	0.33066 (16)	0.04249 (11)	0.0166 (3)	
C1	0.98097 (18)	0.15055 (17)	0.09731 (12)	0.0106 (3)	
C2	1.06827 (19)	0.25260 (18)	0.10370 (13)	0.0140 (4)	
H2	1.1417	0.2527	0.0680	0.017*	
C3	1.04872 (19)	0.35431 (19)	0.16189 (13)	0.0158 (4)	
H3	1.1089	0.4236	0.1651	0.019*	
C4	0.94235 (19)	0.35675 (19)	0.21573 (13)	0.0148 (4)	
C5	0.85693 (19)	0.25278 (19)	0.21028 (13)	0.0142 (4)	
H5	0.7847	0.2514	0.2471	0.017*	
C6	0.87603 (18)	0.15088 (18)	0.15165 (12)	0.0136 (4)	
H6	0.8167	0.0809	0.1488	0.016*	
C7	0.9200 (2)	0.46954 (19)	0.27623 (15)	0.0204 (4)	
H7A	0.8970	0.5443	0.2377	0.031*	
H7B	1.0004	0.4873	0.3160	0.031*	
H7C	0.8482	0.4506	0.3144	0.031*	
C8	0.2184 (2)	0.6093 (2)	0.02768 (15)	0.0249 (5)	
H8	0.1593	0.6526	−0.0157	0.030*	
C9	0.2777 (2)	0.5010 (2)	0.00204 (15)	0.0201 (4)	
H9	0.2596	0.4687	−0.0586	0.024*	
C10	0.36716 (18)	0.43551 (18)	0.06587 (13)	0.0131 (4)	
C11	0.3857 (2)	0.48772 (18)	0.15624 (14)	0.0171 (4)	
H11	0.4421	0.4461	0.2022	0.021*	
C12	0.3229 (2)	0.5969 (2)	0.17697 (14)	0.0202 (4)	
H12	0.3367	0.6314	0.2373	0.024*	
C13	0.4097 (2)	0.2765 (2)	−0.05001 (14)	0.0208 (4)	
H13A	0.3200	0.2431	−0.0599	0.031*	
H13B	0.4726	0.2072	−0.0565	0.031*	
H13C	0.4225	0.3430	−0.0956	0.031*	
C14	0.5171 (2)	0.2619 (2)	0.11039 (15)	0.0249 (5)	
H14A	0.5837	0.3204	0.1387	0.037*	
H14B	0.5603	0.1920	0.0801	0.037*	
H14C	0.4653	0.2270	0.1580	0.037*	

### Atomic displacement parameters (Å^2^)

**Table:**

	*U*^11^	*U*^22^	*U*^33^	*U*^12^	*U*^13^	*U*^23^
Sn1	0.01059 (9)	0.00929 (9)	0.01105 (9)	0.00024 (6)	−0.00081 (6)	−0.00130 (6)
Br1	0.01585 (10)	0.01282 (10)	0.01285 (9)	−0.00079 (7)	0.00140 (7)	0.00179 (7)
Br2	0.01007 (10)	0.01519 (10)	0.02186 (11)	0.00061 (7)	−0.00312 (7)	−0.00193 (7)
N1	0.0260 (10)	0.0161 (9)	0.0291 (10)	0.0075 (7)	0.0021 (8)	0.0008 (7)
N2	0.0155 (8)	0.0160 (8)	0.0179 (8)	0.0010 (7)	−0.0002 (6)	0.0011 (7)
C1	0.0124 (9)	0.0099 (8)	0.0091 (8)	0.0006 (7)	−0.0018 (7)	−0.0002 (7)
C2	0.0120 (9)	0.0134 (9)	0.0165 (9)	−0.0010 (7)	0.0013 (7)	−0.0003 (7)
C3	0.0139 (9)	0.0119 (9)	0.0210 (10)	−0.0036 (7)	−0.0009 (7)	−0.0015 (7)
C4	0.0157 (9)	0.0137 (9)	0.0143 (9)	0.0041 (8)	−0.0027 (7)	−0.0022 (7)
C5	0.0119 (9)	0.0185 (10)	0.0124 (9)	0.0022 (7)	0.0024 (7)	0.0002 (7)
C6	0.0126 (9)	0.0143 (9)	0.0136 (9)	−0.0019 (7)	−0.0006 (7)	0.0005 (7)
C7	0.0218 (11)	0.0158 (10)	0.0232 (10)	0.0042 (8)	0.0005 (8)	−0.0068 (8)
C8	0.0252 (11)	0.0265 (12)	0.0225 (11)	0.0073 (9)	−0.0016 (9)	0.0083 (9)
C9	0.0193 (11)	0.0246 (11)	0.0157 (10)	0.0023 (8)	−0.0021 (8)	0.0035 (8)
C10	0.0110 (9)	0.0119 (9)	0.0165 (9)	−0.0037 (7)	0.0011 (7)	0.0020 (7)
C11	0.0153 (10)	0.0175 (10)	0.0177 (10)	−0.0019 (8)	−0.0035 (8)	0.0019 (8)
C12	0.0208 (11)	0.0179 (10)	0.0214 (10)	−0.0019 (8)	−0.0012 (8)	−0.0016 (8)
C13	0.0203 (11)	0.0228 (11)	0.0194 (10)	−0.0016 (9)	0.0025 (8)	−0.0038 (8)
C14	0.0275 (12)	0.0205 (11)	0.0259 (11)	0.0108 (9)	−0.0016 (9)	0.0030 (9)

### Geometric parameters (Å, °)

**Table:**

Sn1—C1^i^	2.1424 (18)	C5—C6	1.393 (3)
Sn1—C1	2.1424 (18)	C5—H5	0.9500
Sn1—Br2^i^	2.7349 (2)	C6—H6	0.9500
Sn1—Br2	2.7349 (2)	C7—H7A	0.9800
Sn1—Br1	2.76515 (18)	C7—H7B	0.9800
Sn1—Br1^i^	2.76515 (18)	C7—H7C	0.9800
N1—C12	1.346 (3)	C8—C9	1.356 (3)
N1—C8	1.346 (3)	C8—H8	0.9500
N1—H1	0.8800	C9—C10	1.422 (3)
N2—C10	1.335 (2)	C9—H9	0.9500
N2—C14	1.459 (3)	C10—C11	1.424 (3)
N2—C13	1.462 (3)	C11—C12	1.359 (3)
C1—C6	1.388 (3)	C11—H11	0.9500
C1—C2	1.391 (3)	C12—H12	0.9500
C2—C3	1.388 (3)	C13—H13A	0.9800
C2—H2	0.9500	C13—H13B	0.9800
C3—C4	1.396 (3)	C13—H13C	0.9800
C3—H3	0.9500	C14—H14A	0.9800
C4—C5	1.394 (3)	C14—H14B	0.9800
C4—C7	1.504 (3)	C14—H14C	0.9800
			
C1^i^—Sn1—C1	180.00 (13)	C1—C6—C5	120.63 (18)
C1^i^—Sn1—Br2^i^	89.88 (5)	C1—C6—H6	119.7
C1—Sn1—Br2^i^	90.12 (5)	C5—C6—H6	119.7
C1^i^—Sn1—Br2	90.12 (5)	C4—C7—H7A	109.5
C1—Sn1—Br2	89.88 (5)	C4—C7—H7B	109.5
Br2^i^—Sn1—Br2	180.000 (12)	H7A—C7—H7B	109.5
C1^i^—Sn1—Br1	89.22 (5)	C4—C7—H7C	109.5
C1—Sn1—Br1	90.78 (5)	H7A—C7—H7C	109.5
Br2^i^—Sn1—Br1	91.340 (6)	H7B—C7—H7C	109.5
Br2—Sn1—Br1	88.660 (6)	N1—C8—C9	121.3 (2)
C1^i^—Sn1—Br1^i^	90.78 (5)	N1—C8—H8	119.3
C1—Sn1—Br1^i^	89.22 (5)	C9—C8—H8	119.3
Br2^i^—Sn1—Br1^i^	88.660 (6)	C8—C9—C10	120.1 (2)
Br2—Sn1—Br1^i^	91.340 (6)	C8—C9—H9	120.0
Br1—Sn1—Br1^i^	180.000 (10)	C10—C9—H9	120.0
C12—N1—C8	120.94 (19)	N2—C10—C9	121.88 (18)
C12—N1—H1	119.5	N2—C10—C11	121.65 (18)
C8—N1—H1	119.5	C9—C10—C11	116.47 (18)
C10—N2—C14	120.81 (17)	C12—C11—C10	120.16 (19)
C10—N2—C13	121.38 (17)	C12—C11—H11	119.9
C14—N2—C13	117.71 (17)	C10—C11—H11	119.9
C6—C1—C2	118.88 (17)	N1—C12—C11	121.0 (2)
C6—C1—Sn1	119.98 (13)	N1—C12—H12	119.5
C2—C1—Sn1	121.04 (13)	C11—C12—H12	119.5
C3—C2—C1	120.37 (17)	N2—C13—H13A	109.5
C3—C2—H2	119.8	N2—C13—H13B	109.5
C1—C2—H2	119.8	H13A—C13—H13B	109.5
C2—C3—C4	121.28 (18)	N2—C13—H13C	109.5
C2—C3—H3	119.4	H13A—C13—H13C	109.5
C4—C3—H3	119.4	H13B—C13—H13C	109.5
C5—C4—C3	117.91 (17)	N2—C14—H14A	109.5
C5—C4—C7	121.42 (17)	N2—C14—H14B	109.5
C3—C4—C7	120.66 (18)	H14A—C14—H14B	109.5
C6—C5—C4	120.90 (17)	N2—C14—H14C	109.5
C6—C5—H5	119.5	H14A—C14—H14C	109.5
C4—C5—H5	119.5	H14B—C14—H14C	109.5
			
C1^i^—Sn1—C1—C6	0.00 (18)	C7—C4—C5—C6	177.77 (18)
Br2^i^—Sn1—C1—C6	44.67 (14)	C2—C1—C6—C5	1.2 (3)
Br2—Sn1—C1—C6	−135.33 (14)	Sn1—C1—C6—C5	−175.23 (14)
Br1—Sn1—C1—C6	−46.67 (14)	C4—C5—C6—C1	0.1 (3)
Br1^i^—Sn1—C1—C6	133.33 (14)	C12—N1—C8—C9	−1.1 (3)
C1^i^—Sn1—C1—C2	0.00 (7)	N1—C8—C9—C10	−0.4 (3)
Br2^i^—Sn1—C1—C2	−131.72 (15)	C14—N2—C10—C9	−177.10 (19)
Br2—Sn1—C1—C2	48.28 (15)	C13—N2—C10—C9	−0.9 (3)
Br1—Sn1—C1—C2	136.94 (15)	C14—N2—C10—C11	2.9 (3)
Br1^i^—Sn1—C1—C2	−43.06 (15)	C13—N2—C10—C11	179.12 (18)
C6—C1—C2—C3	−1.5 (3)	C8—C9—C10—N2	−178.0 (2)
Sn1—C1—C2—C3	174.92 (14)	C8—C9—C10—C11	2.0 (3)
C1—C2—C3—C4	0.5 (3)	N2—C10—C11—C12	177.86 (18)
C2—C3—C4—C5	0.9 (3)	C9—C10—C11—C12	−2.1 (3)
C2—C3—C4—C7	−178.05 (19)	C8—N1—C12—C11	1.0 (3)
C3—C4—C5—C6	−1.1 (3)	C10—C11—C12—N1	0.7 (3)

Symmetry codes: (i) −*x*+2, −*y*, −*z*.

### Hydrogen-bond geometry (Å, °)

**Table:**

*D*—H···*A*	*D*—H	H···*A*	*D*···*A*	*D*—H···*A*
N1—H1···Br1^ii^	0.88	2.75	3.448 (2)	138
N1—H1···Br2^ii^	0.88	2.94	3.517 (2)	125

Symmetry codes: (ii) *x*−1, *y*+1, *z*.
